# WISP-1, a novel angiogenic regulator of the CCN family, promotes oral squamous cell carcinoma angiogenesis through VEGF-A expression

**DOI:** 10.18632/oncotarget.2978

**Published:** 2015-01-30

**Authors:** Jing-Yuan Chuang, Po-Chun Chen, Ching-Wen Tsao, An-Chen Chang, Ming-Yu Lein, Ching-Chia Lin, Shih-Wei Wang, Chiao-Wen Lin, Chih-Hsin Tang

**Affiliations:** ^1^ Department of Medical Laboratory Science and Biotechnology, China Medical University, Taichung, Taiwan; ^2^ Graduate Institute of Basic Medical Science, China Medical University, Taichung, Taiwan; ^3^ Department of Medical Research, Chung Shan Medical University Hospital, Chung Shan Medical University, Taichung, Taiwan; ^4^ Division of Hematology and Oncology, Department of Internal Medicine, China Medical University Hospital, Taichung, Taiwan; ^5^ Department of Veterinary Medicine, National Chung Hsing University, Taichung, Taiwan; ^6^ Department of Medicine, Mackay Medical College, New Taipei City, Taiwan; ^7^ Institute of Oral Sciences, Chung Shan Medical University, Taichung, Taiwan; ^8^ Department of Dentistry, Chung Shan Medical University Hospital, Taichung, Taiwan; ^9^ Department of Pharmacology, School of Medicine, China Medical University, Taichung, Taiwan; ^10^ Department of Biotechnology, College of Health Science, Asia University, Taichung, Taiwan

**Keywords:** WISP-1, VEGF-A, oral squamous cell carcinoma, angiogenesis

## Abstract

Oral squamous cell carcinoma (OSCC), which accounts for nearly 90% of head and neck cancers, is characterized by poor prognosis and a low survival rate. VEGF-A is the most established angiogenic factor involved in the angiogenic-regulated tumor progression. WISP-1/CCN4 is an extracellular matrix-related protein that belongs to the Cyr61, CTGF, Nov (CCN) family and regulates many biological functions, such as angiogenesis. Previous studies indicated the role of WISP-1 in tumor progression. However, the angiogenic property of WISP-1 in the cancer microenvironment has never been discussed. Here, we provide novel insights regarding the role of WISP-1 in the angiogenesis through promoting VEGF-A expression. In this study, the correlation of WISP-1 and VEGF-A was confirmed by IHC staining of specimens from patients with OSCC. *In vitro* results indicated that WISP-1 induced VEGF-A expression via the integrin αvβ3/FAK/c-Src pathway, which transactivates the EGFR/ERK/HIF1-α signaling pathway in OSCC. This pathway in turn induces the recruitment of endothelial progenitor cells and triggers the neovascularization in the tumor microenvironment. Our *in vivo* data revealed that tumor-secreted WISP-1 promoted the angiogenesis through VRGF expression and increased angiogenesis-related tumor growth. Our study offers new information that highlights WISP-1 as a potential novel therapeutic target for OSCC.

## INTRODUCTION

Oral cancer is the one of the most common cause of cancer-related death. More than 90% of oral malignancies are oral squamous cell carcinomas (OSCC) [1–3]. About half of the patients with OSCC present with lymph nodes metastasis at the time of diagnosis. OSCC has poor prognosis and reduced survival rates [4]. To date, current therapy remains inadequate and prognosis is poor, with the 5-year survival rate being approximately 50% [5, 6].

Cancer cells have the potential for extreme high and unlimited growth [7]. A hypoxic microenvironment is common and leads to local and systemic cancer progression, resistance to therapy, and poor outcome [8, 9]. Among the hypoxia-regulated genes, the vascular endothelial growth factor-A (VEGF-A) is a crucial regulatory factor of angiogenesis in the adaptation of cells to a hypoxic microenvironment [10]. VEGF-A is a multifunctional cytokine. Its biological function is dominantly associated with endothelial cells and promotes angiogenesis. Increasing levels of VEGF-A expression are discussed in many different cancers, including OSCC [11, 12].

WNT1-inducible signaling pathway protein 1 (WISP-1/CCN4) is a cysteine-rich protein that belongs to the Cyr61, CTGF, Nov (CCN) family of matricellular proteins, which have developmental functions [13, 14]. CCN proteins are mostly secreted and are associated to the extracellular matrix (ECM), which has been demonstrated to play important roles in tumor development, including tumor survival, proliferation, migration, and invasion [15, 16]. They may connect signaling pathways and facilitate crosstalk between the epithelium and stroma [13]. It has been reported that overexpression of WISP-1 in normal rat kidney fibroblasts promotes their transformation [17]. WISP-1 is expressed in developing breast tumors in transgenic mice [18]. Moreover, increasing evidence suggests that WISP-1 enhances tumorigenesis and metastasis in many types of cancer [19, 20]. These data suggest that WISP-1 plays a critical role during cancer development and metastasis.

A previous study indicates that VEGF-A is the most important angiogenic factor while participating in tumorigenesis [21]. Correlation between VEGF-A expression and OSCC progression has been previously discussed [11, 12]. Moreover, our previous work showed that WISP-1 is associated with OSCC cell migration [22]. Many members of the CCN family have been proposed to exert angiogenic functions [23, 24], but WISP-1 was never reported as one of them. Here, we provide evidence that WISP-1 promotes OSCC tumorigenesis through VEGF-A-promoted angiogenesis. WISP-1 expression was correlated with VEGF-A expression and tumor stage in OSCC specimens. Treatment of OSCC cells with WISP-1 induced VEGF-A expression and promoted endothelial progenitor cells (EPCs) recruitment, contributing to neovascularization. We also demonstrated the involvement of the integrin αvβ3/focal adhesion kinase (FAK)/c-Src/epidermal growth factor receptor (EGFR)/extracellular signal-regulated kinase (ERK)/hypoxia inducible factor 1 (HIF-1) α signaling pathway in the VEGF-A-promoted angiogenesis. Pretreatment with VEGF-A neutralizing antibody dramatically reduced this effect. The role of WISP-1 in the VEGF-A-promoted angiogenesis was also confirmed using the chick chorioallantoic membrane assay (CAM) and mouse xenograft *in vivo* models. In summary, our present work indicates that WISP-1 affects OSCC tumorigenesis through VEGF-A-promoted angiogenesis.

## RESULTS

### Clinical significance of WISP-1 and VEGF-A expression in specimens from patients with OSCC

**Figure 1:**
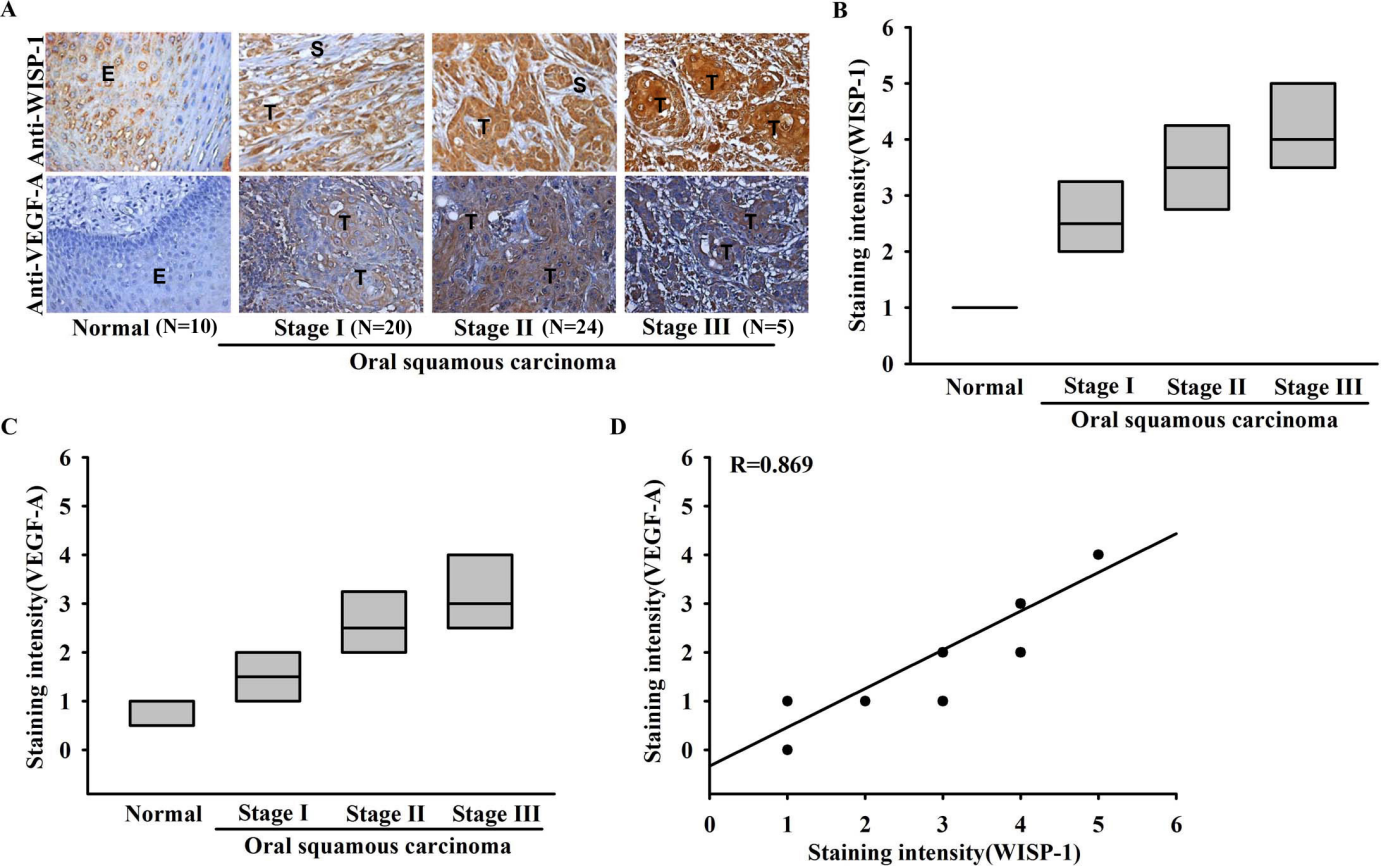
Clinical significance of WISP-1 and VEGF-A in specimens from patients with OSCC Tumor specimens were immunostained (IHC) with anti-WISP-1 and anti-VEGF-A antibodies. The staining intensity was scored 1–5. **(A)** IHC photographs. (E = epithelial, T = tumor, S = stroma). **(B–D)** Quantitative results and correlation between WISP-1, VEGF-A, and OSCC clinical grade.

Our previous study showed that WISP-1 is associated with OSCC cells migration [22]. To investigate the role of WISP-1 in the OSCC angiogenesis, we first examined the expression profile of WISP-1 and VEGF-A in specimens from patients with OSCC using two submitted microarray datasets (GSE3524 and GSE2280) that contain information from 47 patients with OSCC. As shown in Figure S1A and 1B, WISP-1 and VEGF-A expression levels were higher in tumor specimens than in normal tissues. Moreover, their expression levels were also higher in metastatic tumors than in primary tumors (Figure S1C and 1D). Thus, WISP-1 high expression maybe correlated with the angiogenesis in OSCC. To evaluate the correlation between WISP-1 and VEGF-A, WISP-1 and VEGF-A IHC was performed on tissue specimens obtained from 60 patients with OSCC. The IHC results indicated that WISP-1 and VEGF-A were nearly undetectable in normal tongue epithelial cells, but were associated with higher clinical pathologic grade, and expression patterns of WISP-1 and VEGF-A were correlated with tumor stage (Figure 1A–1C). The quantitative data also showed that WISP-1 expression was correlated with VEGF-A expression in human OSCC specimens (Figure 1D). Moreover, our result indicated that OSCC cell lines (SCC4 and SAS) and OSCC tumor specimens showed highly expression of VEGF-A protein compared with normal specimens (Figure S2). These results suggest that WISP-1 is associated with VEGF-A expression and tumor progression in patients with OSCC.

### WISP-1 regulates angiogenesis by increasing VEGF-A expression in OSCC cells

**Figure 2:**
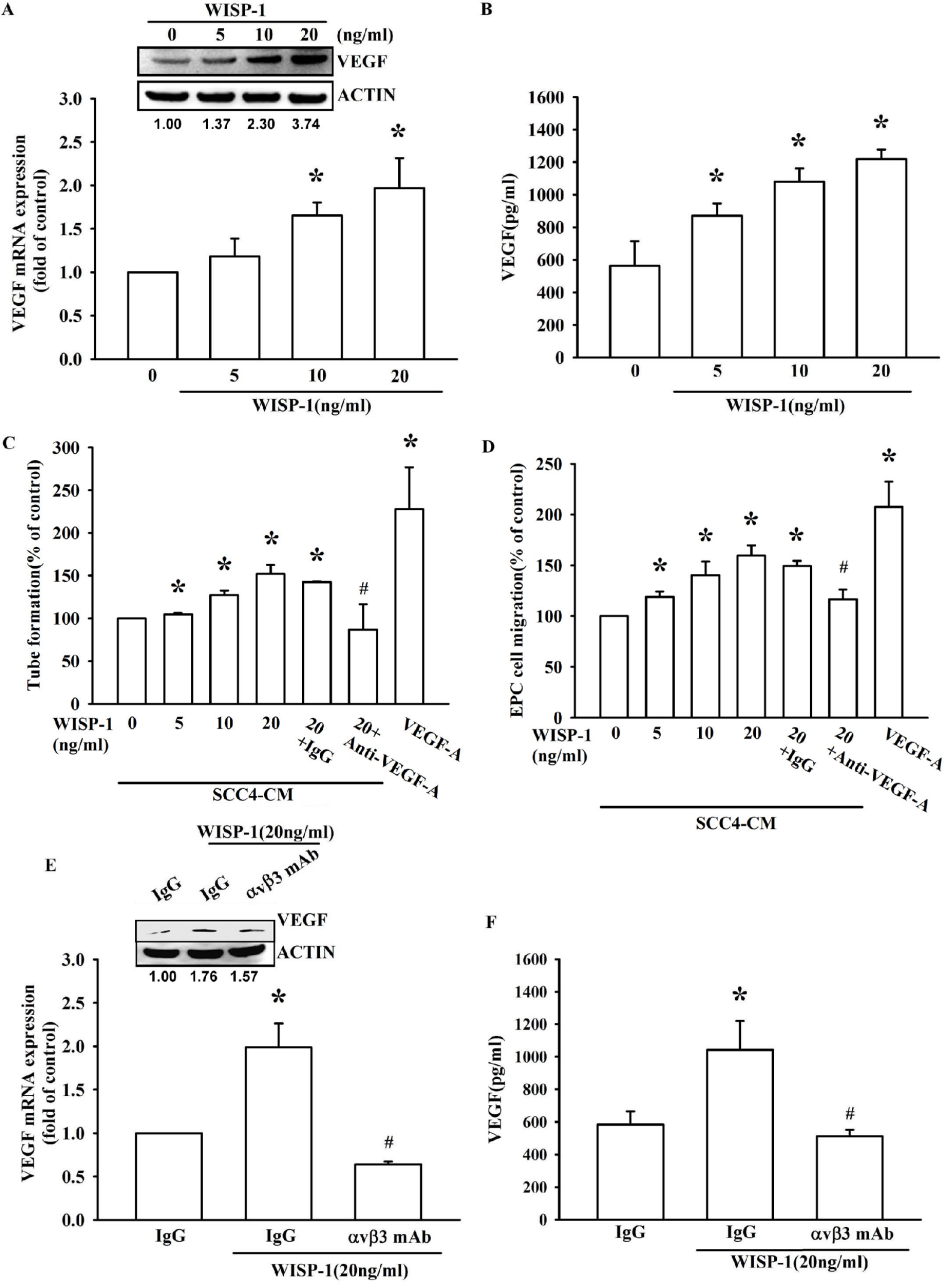
WISP-1 regulates the angiogenesis by raising VEGF-A expression in OSCC cells **(A–B)** SCC4 cells were incubated with WISP-1 (0–20 ng/mL) for 24 h, VEGF-A expression was measured by qPCR, ELISA, and western blot. **(C–D)** SCC4 cells were incubated with WISP-1 (0–20 ng/mL) for 24 h, and the CM was collected. EPCs were pre-treated for 30 min with IgG control antibody or VEGF-A antibody (1 μg/mL) and incubated with CM for 6 h and cell capillary-like structure formation in EPCs was examined by tube formation assay (C) EPCs were incubated with CM for 24 h, and cell migration was examined using the transwell assay (D) **(E–F)** SCC4 cells were incubated with the integrin αvβ3 antibody for 30 min, followed by stimulation with WISP-1 (20 ng/mL) for 24 h. VEGF-A expression was examined by western blot, qPCR, and ELISA. Data are expressed as mean ± SEM *P < 0.05 compared to control; #*P* < 0.05 compared to the WISP-1 treated group.

Overexpression of VEGF-A has been investigated in many different cancers, including OSCC [11, 12]. Our IHC result indicated that WISP-1 expression is correlated with VEGF-A expression in human OSCC specimens. However, it is important to determine whether WISP-1 promotes VEGF-A expression in OSCC. Our results showed that WISP-1 increased VEGF-A expression and secretion in OSCC cells SCC4 (Figure 2A and 2B) as well as another OSCC cell line SAS (Figure S3A). Moreover, a time-dependently increasing VEGF-A protein expression after WISP-1 treatment has been also certified by western blot (Figure S3B). Previous studies indicate that tumor could recruit EPCs to the tumor microenvironment, inducing their differentiation into endothelial cells and contributing to neovascularization [25]. Transwell migration assay indicated that CM collected from WISP-1-treated OSCC cells increased EPCs migration. In addition, pretreatment with a VEGF-A neutralizing antibody but not IgG isotype antibody abolished this effect (Figure 2C), indicating that EPCs may be recruited to the tumor microenvironment by WISP-1-regulated VEGF-A expression in OSCC cells. We also examined the angiogenic function of the recruited EPCs, and the data indicated that CM collected from WISP-1-treated OSCC cells increased EPCs tube formation, which was inhibited by VEGF-A neutralizing antibody treatment but not IgG isotype antibody (Figure 2D). The angiogenic role of WISP-1 has also been improved in HUVEC cells (Figure S3C). However, when we directly treated WISP-1 alone with EPCs, it didn’t induce tube formation (Figure S4). These data indicated the WISP-1-promoted angiogenesis in OSCC was VEGF-A dependent. Cell surface receptors are key mediators to coordinate cell response to extracellular signals. Our previous studies indicate that integrin αvβ3 play a crucial role in WISP-1 signaling regulation in different cancer, and the results showed that pretreatment with integrin αvβ3 antibody could abolish WISP-1-promoted cell migration in SCC4 cells. [22, 26]. Therefore, we suggested that integrin αvβ3 may regulate VEGF-A expression by WISP-1 treatment. As expected, pretreatment of OSCC cells with an integrin αvβ3 antibody inhibited WISP-1-induced VEGF-A expression (Figure 2E and 2F). These results reveal that WISP-1 promotes VEGF-A expression through integrin αvβ3, which in turn regulates the angiogenesis within the OSCC microenvironment.

### WISP-1 promotes VEGF-A expression in OSCC cells and contributes to the angiogenesis through the FAK/c-Src signaling pathway

**Figure 3:**
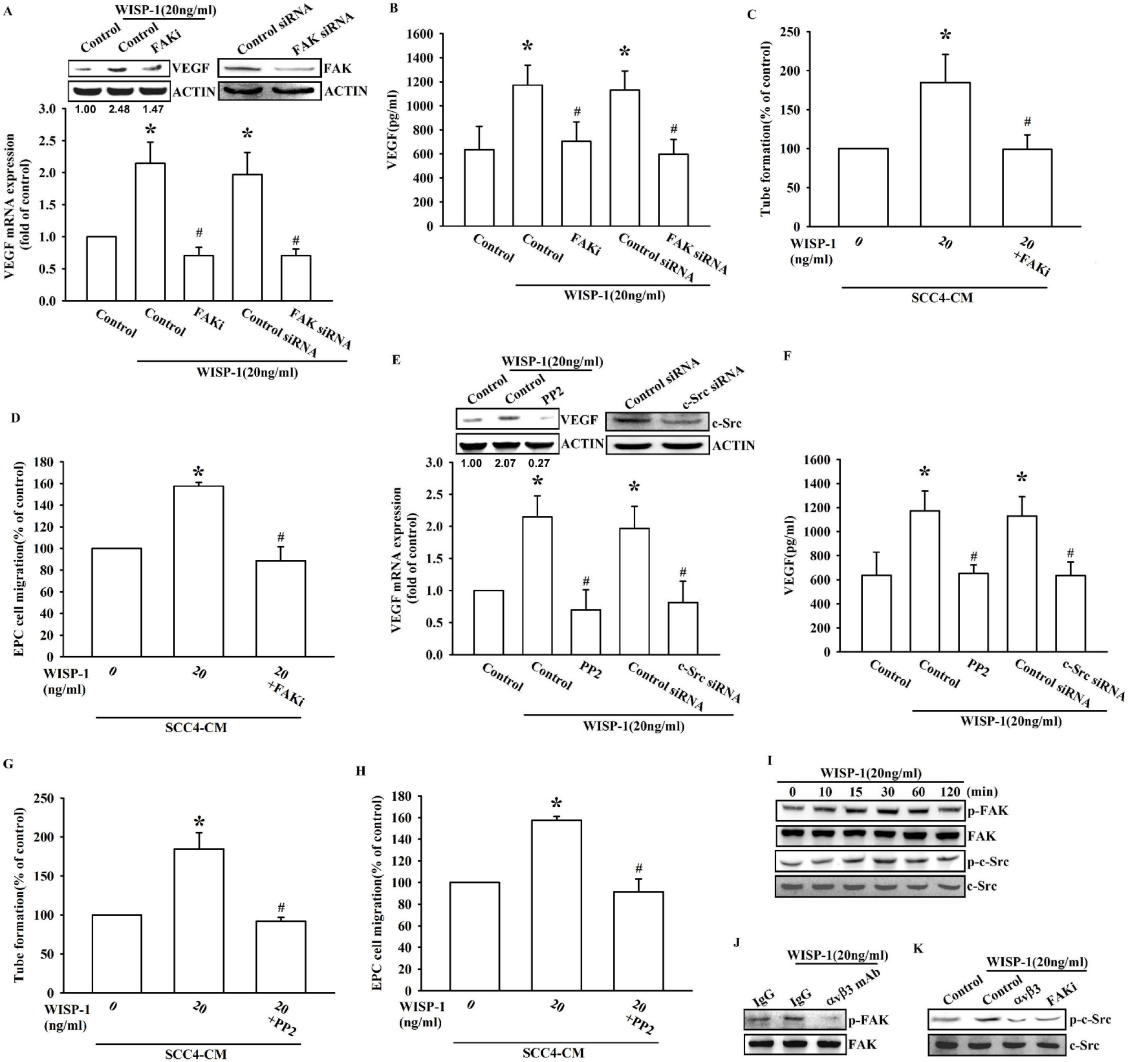
FAK/Src signaling pathway is involved in WISP-1-promoted VEGF-A expression and contributing to angiogenesis **(A–B)** SCC4 cells were pre-treated with a FAK inhibitor (FAKi; 1 μM) for 30 min or transfected with FAK siRNAs for 24 h, followed by WISP-1 (20 ng/mL) stimulation for 24 h. VEGF-A expression was examined by western blot, qPCR, and ELISA. **(C–D)** SCC4 cells were pre-treated with a FAK inhibitor (FAKi; 1 μM), followed by WISP-1 (20 ng/mL) stimulation for 24 h. CM was collected. EPCs were incubated with CM for 6 h and capillary-like structure formation in EPCs was examined by tube formation assay (C) EPCs were incubated with CM for 24 h and cell migration was examined by transwell assay (D) **(E–H)** SCC4 cells were treated with a Src inhibitor (pp2; 1 μM) for 30 min or transfected with Src siRNAs for 24 h, followed by WISP-1 (20 ng/mL) stimulation for 24 h. Assay procedure was performed as in (A–D) **(I)** SCC4 cells were incubated with WISP-1 (20 ng/mL) for the indicated times, and FAK and c-Src phosphorylation was determined by western blot. **(J–K)** SCC4 cells were incubated with an integrin αvβ3 antibody or FAKi for 30 min, followed by stimulation with WISP-1 (20 ng/mL) for 60 min, and FAK (J) and c-Src (K) phosphorylation was determined by western blot. Data are expressed as the mean ± SEM *P < 0.05 compared with control; #*P* < 0.05 compared with the WISP-1-treated group.

Our data indicate that WISP-1 elicits signal transduction through integrin αvβ3. We therefore analyzed signal pathways that are known to be regulated by integrin receptors in OSCC cells. FAK/c-Src dual kinase complex, the common signal modulator downstream of integrin, functions to promote cell motility, cell cycle, and cell survival. It has recently been implicated in tumor progression processes such as angiogenesis and metastasis [27]. Pretreatment with FAK inhibitor or FAK siRNA transfection reversed the WISP-1-induced VEGF-A expression in OSCC cells (Figure 3A and 3B). Furthermore, EPC migration and tube formation, which were induced by WISP-1-treated OSCC cells CM, were also abolished (Figure 3C and 3D). Similarly, pretreatment of OSCC cells with c-Src inhibitor or cSrc siRNA transfection confirmed the involvement of c-Src in WISP-1-induced VEGF-A expression and subsequent angiogenesis function in EPCs (Figure 3E–3H). Finally, pretreatment of OSCC cells with WISP-1 increased the phosphorylation of FAK and c-Src signaling proteins (Figure 3I). We also demonstrated the role of the integrin αvβ3/FAK/c-Src axis in OSCC cells using an integrin αvβ3 antibody and FAK inhibitor. Pretreatment with integrin αvβ3 antibody or FAKi block downstream signal proteins FAK or c-Src activation respectively (Figure 3J–3K). These results indicate that WISP-1 regulates VEGF-A expression in OSCC cells and contributing to angiogenesis through the FAK/c-Src signaling pathway.

### WISP-1 elicits EGFR transactivation and downstream effector ERK activation, in turn promoting VEGF-A expression and angiogenesis in OSCC cells

**Figure 4:**
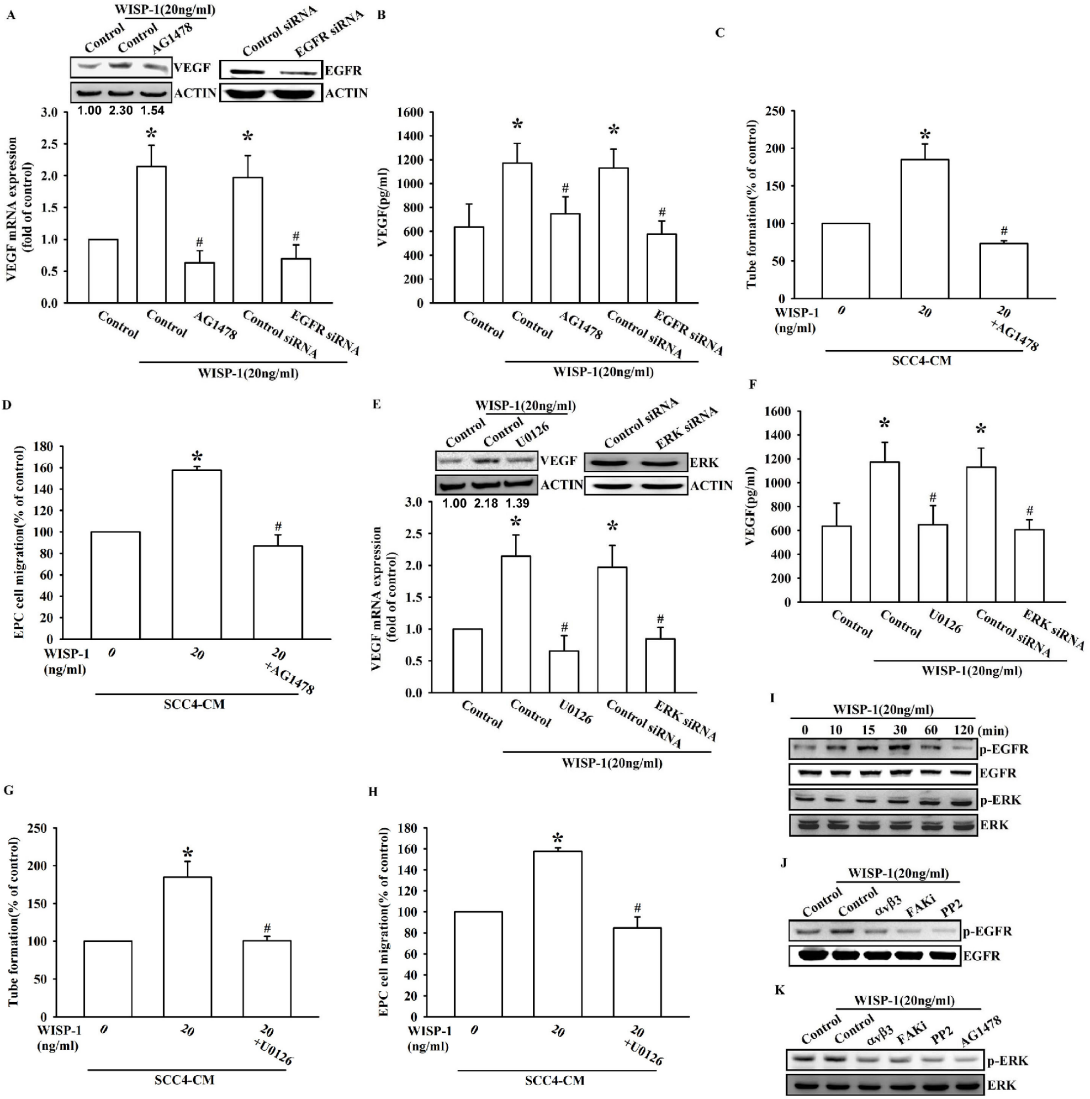
EGFR transactivation is involved in WISP-1-induced VEGF-A expression and contributing to angiogenesis **(A–D)** SCC4 cells were pre-treated with an EGFR inhibitor (AG1478; 1 μM) for 30 min or transfected with EGFR siRNAs for 24 h, followed by WISP-1 (20 ng/mL) stimulation for 24 h. The assay procedures were performed as described in Figure 3A–3D. **(E–H)** SCC4 cells were treated by an ERK inhibitor (U0126; 1 μM) for 30 min or transfected with ERK siRNAs for 24 h, followed by WISP-1 (20 ng/mL) stimulation for 24 h. The assay procedures were performed as described in Figure 3A–3D. **(I)** SCC4 cells were incubated with WISP-1 (20 ng/mL) for the indicated times and EGFR and ERK phosphorylation was determined by western blot. **(J–K)** SCC4 cells were incubated with the integrin αvβ3 antibody, FAKi, PP2, or AG1478 for 30 min, followed by stimulation with WISP-1 (20 ng/mL) for 60 min, and EGFR (J) and ERK (K) phosphorylation was determined by western blot. Data are expressed as the mean ± SEM *P < 0.05 compared with control; #*P* < 0.05 compared with the WISP-1-treated group.

WISP-1 is an ECM-associated protein, which interacts with the integrin family of cell-surface receptors. A previous study reported that integrin, the most important cell-surface receptor without intrinsic enzymatic activity, could elicit EGFR transactivation in order to generate further cellular responses [28]. Moreover, enormous reports indicate that Src protein play a crucial role in EGFR transactivation [29, 30] and other study shows that integrin β3 elicits Src-mediated EGFR transactivation [31]. However, whether CCN protein could elicit signal transduction through the integrin/EGFR transactivation axis has never been discussed. Pretreatment with an EGFR inhibitor or EGFR siRNA transfection reversed the WISP-1-induced VEGF-A expression in OSCC cells (Figure 4A and 4B) as well as EPC migration and tube formation (Figure 4C and 4D). We also determined whether ERK, the canonical effector downstream of EGFR, was involved in WISP-1-induced VEGF-A expression. The involvement of ERK in WISP-1-induced VEGF-A expression and subsequent angiogenesis function in EPCs was examined by EKR inhibitor pretreatment or siRNA transfection in OSCC cells (Figure 4E–4H). The activation of EGFR and ERK was also confirmed by increase in the levels of phosphorylated EGFR and ERK proteins in WISP-1-treated OSCC cells (Figure 4I). At last, we identified that the integrin αvβ3/FAK/c-Src signaling cascade mediated EGFR transactivation, and in turn ERK activation (Figure 4J–4K). These results indicate that WISP-1 regulates VEGF-A expression in OSCC and promoting angiogenesis through integrin αvβ3/FAK/c-Src mediated EGFR transactivation and ERK signaling pathway.

### WISP-1 promotes VEGF-A expression in OSCC cells and the angiogenesis through the HIF1-α signaling pathway

**Figure 5:**
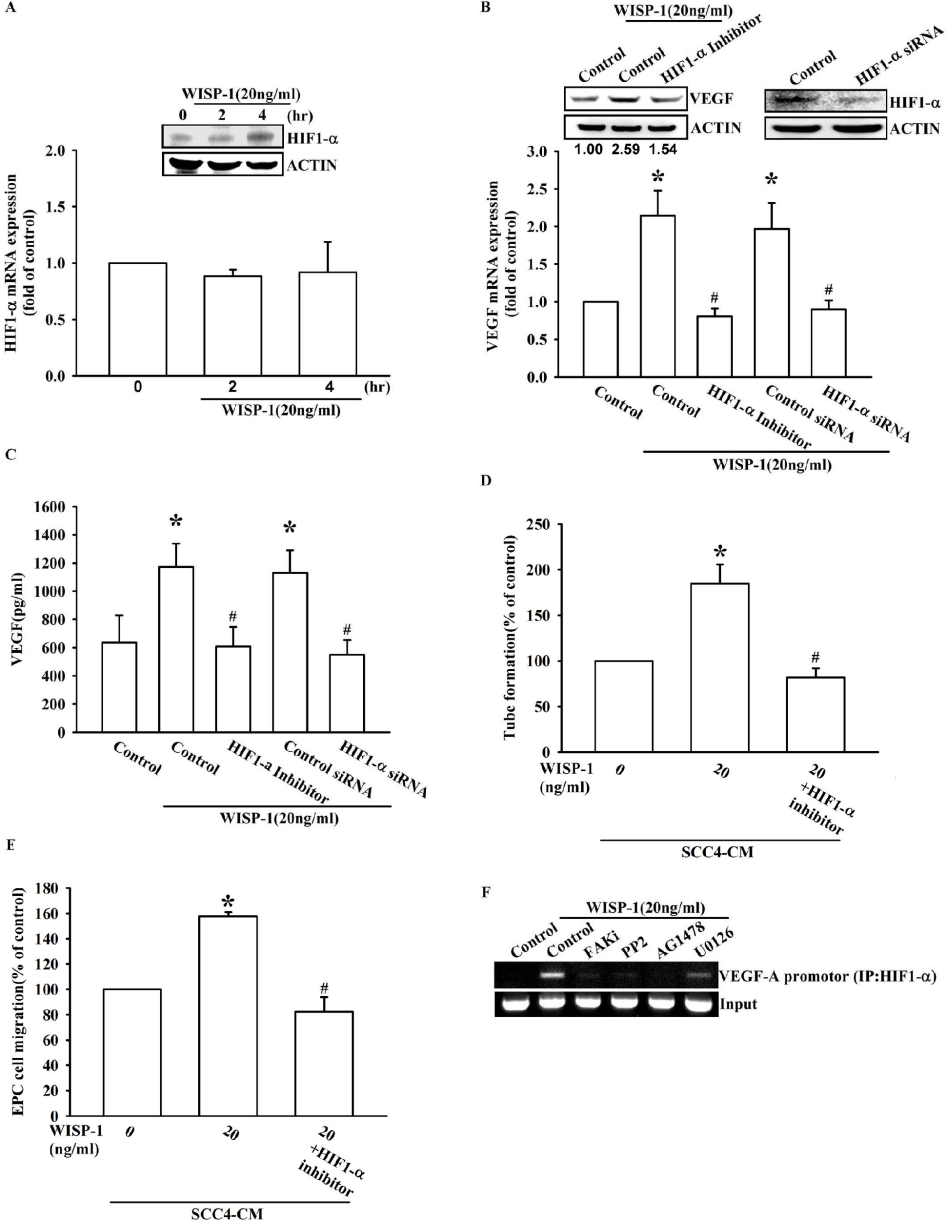
WISP-1 promotes VEGF-A expression in OSCC and contributing to angiogenesis through the HIF1-α signaling pathway **(A)** SCC4 cells were stimulated by WISP-1 (20 ng/mL) for the indicated times (0, 2, and 4 h). HIF1-α expression level was measured by western blot and qPCR. **(B–E)** SCC4 cells were pre-treated with HIF1-α inhibitor (1 μM) for 30 min or transfected with HIF1-α siRNAs for 24 h, followed by WISP-1 (20 ng/mL) stimulation for 24 h. The assay procedures were performed as described in Figure 3A–3D. **(F)** SCC4 cells were incubated with FAKi, PP2, AG1478, or U0126 for 30 min, followed by stimulation with WISP-1 (20 ng/mL) for 60 min. Chromatin immunoprecipitation (ChIP) assays were performed using an anti-HIF1-α antibody. One percent of the precipitated chromatin was analyzed to verify equal loading (input). Data are expressed as the mean ± SEM. *P < 0.05 compared with control; #*P* < 0.05 compared with the WISP-1-treated group.

Hypoxia is common feature in OSCC and contributes to tumor-related angiogenesis. HIF1-α is proposed as a central role in the adaptation to a hypoxic microenvironment [32]. Many reports have documented that HIF-1α is the canonical transcription factor involved in VEGF-A expression [33]. Here, we provide evidence that WISP-1 induces VEGF-A expression through HIF1-α activation. WISP-1 treatment directly increased HIF-1α protein accumulation in a time-dependent manner (Figure 5A, upper panel). However, mRNA expression of HIF-1α was invariable (Figure 5A, lower panel). This phenomenon revealed that WISP-1 treatment improved HIF-1α protein stability in OSCC cells. HIF-1α inhibitor pretreatment or siRNA transfection reversed the WISP-1-induced VEGF-A expression in OSCC cells (Figure 5B and 5C). As expected, EPCs migration and tube formation, induced by WISP-1-treated OSCC cells CM, were also abolished (Figure 5D and 5E). HIF-1α activation was further evaluated by ChIP assay analysis. The results indicated that pretreatment of OSCC cells with FAK, Akt, EGFR, and ERK inhibitors decreased HIF-1α binding to its DNA binding site (Figure 5F). These results indicate that WISP-1 regulates VEGF-A expression in OSCC and promoting angiogenesis through the HIF-1α transcription factor activation.

### Knockdown of WISP-1 expression decreases VEGF-A expression and inhibits angiogenesis in OSCC cells

**Figure 6:**
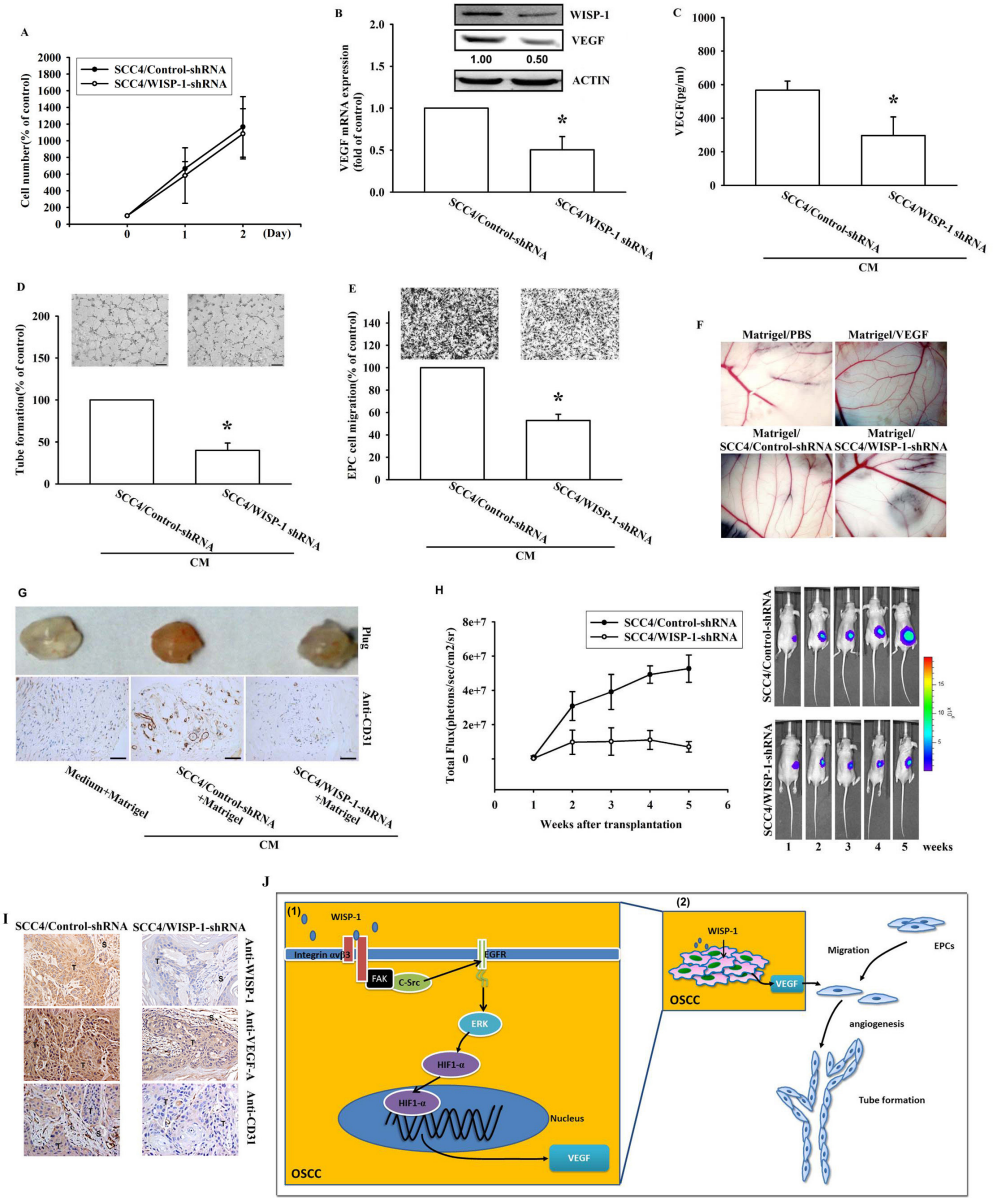
WISP-1 knockdown in OSCC decreases VEGF-A expression and angiogenesis-related tumor growth *in vivo* **(A)** SCC4 cells stably expressing shRNA constructs or control shRNA were seeded as monolayers and counted daily. Cells (10^3^) were plated in 6 well plates and grown for 2 days. Cells were trypsinized, and cell numbers was counted. **(B–C)** WISP-1 and VEGF-A mRNA and protein expression in SCC4 cells stably expressed a control shRNA or a WISP-1 shRNA was examined by western blot, qPCR, and ELISA. **(D–E)** EPCs were incubated with CM collected from control-shRNA and WISP-1-shRNA transfected SCC4 cells for 24 h and cell migration or tube formation were examined. **(F)** PBS, VEGF-A, control shRNA/SCC4 CM, and WISP-1 shRNA/SCC4 CM mixed in Matrigel were placed on chick chorioallantoic membranes. CAMs in each group were photographed on developmental day 12. **(G)** Mice were subcutaneously injected with Matrigel mixed with PBS, control shRNA/SCC4 CM or WISP-1 shRNA/SCC4 CM for seven days. Plugs excised from the mice were photographed and stained with CD31. **(H)** Control shRNA and WISP-1 shRNA SCC4 cells were mixed with Matrigel and injected into the flank of the mice for 28 days. Tumor growth was monitored using the IVIS Imaging System. Tumor growth was quantified by fluorescent imaging from week 0–6. **(I)** Tumors were paraffin embedded, and sections were immunostained using the WISP-1, VEGF-A, and CD31 antibodies. (E = epithelial, T = tumor, S = stroma). **(J)** Diagrammatic model for the role of WISP-1 in OSCC. (1) WISP-1 induces VEGF-A expression and secretion in OSCC cells through the integrin αvβ3/FAK/c-Src pathway, which transactivates the EGFR/ERK/HIF1-α signal pathway. (2) The WISP-1-induced secretion of VEGF-A subsequently recruiting EPCs to OSCC tumor microenvironment and promoting neoangiogenesis. Data represent the mean ± SEM *P < 0.05 compared to control shRNA/SCC4.

Our results indicated that WISP-1 promoted VEGF-A expression and enhanced angiogenesis by recruiting EPCs and increasing tube formation in the microenvironment. Thus, we investigated the role of WISP-1 *in vivo*. To confirm its regulatory role in VEGF-A expression, we utilized OSCC cells stably expressing a WISP-1 shRNA. Knockdown of WISP-1 expression in OSCC cells didn’t affect cell proliferation (Figure 6A). Results showed that WISP-1 and VEGF-A expression levels were decreased in WISP-1 shRNA transfected OSCC cells (Figure 6B and 6C). CM collected from OSCC cells stably expressing a control-shRNA promoted EPC cell migration and tube formation, while CM collected from OSCC cells stably expressing WISP-1 shRNA decreased EPC cell migration and tube formation (Figure 6D and 6E). Finally, the role of WISP-1, *in vivo*, was examined by chick embryo chorioallantoic membrane (CAM) assay. As expected, CM collected from OSCC cells stably expressing a control shRNA enhanced CAM angiogenesis, while CM from WISP-1 shRNA cells completely reduced angiogenesis in CAMs (Figure 6F). The results of the *in vivo* Matrigel plug formation assay by subcutaneous implantation in mice showed that Matrigel mixed with CM from control-shRNA transfected SCC4 cells increased blood vessel growth, while CM from WISP-1 shRNA transfected SCC4 cells reduced neovascularization (Figure 6G, upper panel). CD31 IHC and hemoglobin content assay indicated a decline in vascular formation in Matrigel (Figure 6G, lower panel). These results indicate that WISP-1 promotes the angiogenesis *in vivo*.

### Knockdown of WISP-1 decreases angiogenesis-related tumor growth *in vivo*

To evaluate whether WISP-1 contributes to VEGF-A expression and in turns to promote angiogenesis which inducing tumor growth *in vivo*, the control-shRNA and WISP-1 shRNA stable SCC4 cell lines were implanted in a mouse xenograft model. Bioluminescence images indicated that control-shRNA transfected SCC4 cells profoundly induced tumor mass formation, but WISP-1 knockdown reduced tumor growth in mice (Figure 6H and Figure S5A–S5C). The angiogenesis level was quantified by examining the plug hemoglobin content and indicated that WISP-1 knockdown impeded OSCC-related angiogenesis *in vivo* (Figure S5D). Hemoglobin content also positively correlated with tumor volume (Figure S5E). Finally, WISP-1 and CD31 IHC results confirmed that WISP-1-promoted the angiogenesis in OSCC tumors (Figure 6I). In summary, these data revealed that WISP-1 promotes the angiogenesis and tumor growth *in vivo*.

## DISCUSSION

The concept of angiogenesis in the tumor microenvironment is extremely important in many tumors, including OSCC. Tumor must develop new blood vessels in order to continue to expand and metastasize. VEGF-A has been implicated as a pivotal target in anti-angiogenic therapy [34]. The effect of WISP-1 on OSCC cell migration ability has been previously discussed [22]. Here, we provide novel insights on the role of WISP-1 in the tumor angiogenesis. The IHC results on clinical specimens from patients with OSCC showed that WISP-1 and VEGF-A expression levels were positively correlated with tumor stage in OSCC. Additionally, WISP-1 regulated VEGF-A expression through the integrin αvβ3/FAK/c-Src mediated EGFR transactivation/ERK/HIF1-α signaling pathway in OSCC cells (Figure 6J). In summary, our study using clinical specimens, cellular experiments, and animal models suggest that WISP-1 regulates VEGF-A expression and induces angiogenesis in OSCC.

The ECM-associated proteins that belong to the CCN family play critical roles in skeletal development, wound healing, fibrosis, and cancer. CCN1, CCN2, and CCN3 have been associated with angiogenesis [35]. However, the role of WISP-1/CCN4 in angiogenesis has not been discussed yet. Our results revealed that WISP-1 induced VEGF-A expression, which in turn promotes angiogenesis in the OSCC microenvironment (Figure 2A and 2B). Our data suggest that angiogenesis-promoted feature of WISP-1 as well as other CCN family proteins through regulation of VEGF-A expression. The multifunctional roles of the CCN family may provide opportunities to develop novel targets for OSCC therapy.

Integrin αvβ3 is known to exert both pro- and anti-angiogenic function [36]. The role of integrin αvβ3 in angiogenesis is defined by its interaction with different receptors or ligands. A previous study indicates that binding of integrin αvβ3 to Cyr61/CCN1, another member of the CCN family, could promote angiogenesis [37]. In this report, we show evidence that WISP-1, as Cyr61, activates integrin αvβ3. Our data indicated that WISP-1 promotes VEGF-A expression, which, in turn, regulates the angiogenesis by integrin αvβ3 (Figure 2E and 2F). Consistent with our previous study, WISP-1 increases cell migration by integrin αvβ3 dependent pathway [22]. These results showed the pivotal role of integrin αvβ3 in WISP-1 signal transduction. However, the direct interaction between integrin αvβ3 and CCN proteins should be further investigated in the future.

Integrins can elicit signal transduction and change cell function through the recruitment and activation of signaling proteins such as non-receptor tyrosine kinases, FAK and c-Src that form a dual kinase complex. The highly activated FAK-Src complex is found in many tumors and activates signals, leading to tumor growth and metastasis [27]. Moreover, a novel role for the FAK-Src complex in angiogenesis has been shown in a mouse model [38]. Another study shows that the FAK/Src kinase complex activates ERK signaling and regulates VEGF-A secretion in breast cancer [39]. Our data are in agreement with previous studies. Through binding to integrin αvβ3, WISP-1 activated the FAK/Src signaling cascade to regulate VEGF-A expression (Figure 3). Interestingly, we identify a novel signal transduction pathway by which WISP-1/integrin αvβ3 interaction elicits EGFR transactivation through FAK/Src activation (Figure 4). Integrin has been shown to elicit EGFR transactivation in order to generate further cellular responses [28]. However, the role of a CCN protein in this pathway has never been reported. Our results bring novel insights on the CCN family proteins signaling.

Until the 1960s, preliminary evidence revealed that tumor-related angiogenesis was mediated by secreted factors produced by cancer cells and promotes tumor progression [40, 41]. Among the numerous factors involved in the angiogenesis, VEGF-A is the most prominent. VEGF-A expression has been correlated with OSCC progression [11, 12]. Our previous study also showed the role of WISP-1 in OSCC cell migration and tumor progression [22]. Here, we provide novel evidence that suggests the angiogenic role of WISP-1 in OSCC. Integrin αvβ3, the most crucial cell surface receptor for the CCN family protein, is thought to be involved in pathological angiogenesis because it is highly expressed on activated and proliferating endothelial cells, but nearly undetectable on quiescent endothelial cells [36]. However, some studies indicate a double-edged effect of integrin through binding to various receptors or ligands [37]. The present study clearly indicates the role of the WISP-1/integrin αvβ3 pathway in the angiogenic function through regulation of VEGF-A expression.

In accordance with our previous investigation [22], WISP-1 promotes both migration ability and angiogenesis effect of OSCC. The pivotal molecular events for tumor spread such as cell migration and neoangiogenesis take place in the invasive front of the OSCC [42]. Our reports reveal the important role of WISP-1 in OSCC metastasis. CTGF, another member of CCN family, has been used in clinical treatment of pancreatic cancer in a Phase I clinical trial [43, 44]. Therefore, blocking of WISP-1 function may serve as novel therapeutic targets in OSCC treatment.

## MATERIALS AND METHODS

### Materials

Anti-mouse and anti-rabbit IgG-conjugated horseradish peroxidase, rabbit polyclonal antibodies specific for p-FAK, FAK, p-c-Src, c-Src, p-EGFR, EGFR, p-ERK, ERK, HIF1-α, β-actin, CD31, and WISP-1 were purchased from Santa Cruz Biotechnology (Santa Cruz, CA); VEGF-A antibody was purchased from Abcam (Cambridge, MA, USA). Recombinant human VEGF-A was purchased from R&D Systems (Minneapolis, MN, USA). Dulbecco's modified Eagle's medium (DMEM), F-12 medium, fetal bovine serum (FBS) and all other cell culture reagents were from Gibco-BRL Life Technologies (Grand Island, NY, USA). ON-TARGETplus siRNAs were purchased from Dharmacon Research (Lafayette, CO, USA). All other chemicals were from Sigma-Aldrich (St Louis, MO, USA). The efficacy of all inhibitors were provided in supplementary information (Figure S6). The methods of Transwell migration assay, Western blot analysis, Quantitative real-time PCR, Enzyme-linked immunosorbent assay, Tube formation, Establishment of the WISP-1 knockdown SCC4 cell line and Chick chorioallantoic membrane assay are provided in Supplementary Information.

### Cell culture

The human OSCC cell line SCC4, an epithelial-type cell line derived from a squamous carcinoma of human tongue, was purchased from Bioresource Collection and Research Center (BCRC, Hsinchu, Taiwan). The human OSCC cell line SAS was kindly provided by Dr. Shun-Fa Yang, Chung Shan Medical University. Cells were cultured in complete medium containing DMEM/F-12 medium supplemented with penicillin, streptomycin, and 10% FBS at 37°C in a 5% CO_2_ atmosphere. Human Umbilical Vein Endothelial Cells (HUVEC) were purchased form American Type Culture Collection (ATCC, VA, USA). Cells were culture in complete medium containing F-12K medium supplemented with 0.1 mg/ml heparin; 0.03–0.05 mg/ml endothelial cell growth supplement (ECGS), penicillin, streptomycin, and 10% FBS at 37°C in a 5% CO_2_ atmosphere.

### Data retrieval from online Gene expression omnibus (GEO) databases

OSCC gene expression profile data from 47 patients with OSCC were downloaded from the GEO database (GSE3524, GSE2280). WISP-1 and VEGF-A expression values were collected independently.

### Endothelial progenitor cell culture

Protocol for EPC culture was approved by the Institutional Review Board of Mackay Medical College, New Taipei City, Taiwan (reference number P1000002). All subjects gave informed written consent before enrolling in our study. Peripheral blood (80 mL) was obtained from healthy donors, and mononuclear cells were isolated using the Ficoll-Paque PLUS centrifuge (Amersham Biosciences, Uppsala, Sweden), according to manufacturer's instructions. CD34-positive progenitor cells were isolated from mononuclear cell fraction by CD34 MicroBead kit and MACS Cell Separation System (Miltenyi Biotec, Bergisch Gladbach, Germany). EPCs were maintained and characterized as described previously [45]. Briefly, human CD34-positive EPCs were cultured in MV2 complete medium that contained MV2 basal medium and growth supplement (PromoCell, Heidelberg, Germany) and was also supplemented with 20% defined FBS (HyClone, Logan, UT, USA). Cultures were seeded on 1% gelatin-coated plasticware and maintained at 37°C in a humidified 5% CO_2_ atmosphere.

### Chromatin immunoprecipitation

Chromatin immunoprecipitation (ChIP) assays were performed as described [46]. Chromatin was prepared and incubated with an anti-HIF1-α antibody. DNA was extracted from the immunoprecipitates, purified, and resuspended in H_2_O. Immuno precipitated DNA was amplified by PCR using the following primers: 5′-CCTTTGGGTTTTGCCAGA-3′ and 5′-CCAAGTTTGTGGAGCTGA-3′ [47]. PCR products were resolved by 1.5% agarose gel electrophoresis and visualized by UV light.

### Animal model and imaging

Experimental procedures were approved by the Institutional Animal Care and Use Committee. Male nu/nu mice (6–8 weeks of age) were subcutaneously injected with 5 × 10^5^ OSCC cells resuspended in 100 μL of medium. Tumor growth, local invasion, and metastasis were monitored using the IVIS Imaging System (PerkinElmer Inc, MA, USA).

### Immunohistochemistry (IHC)

Human oral cancer tissue array (OR601a) was purchased from Biomax (Odenton, MD) in the form of 5 μm sections of paraffin-embedded tissue on glass slides. Sections (5-μm thick) of paraffin-embedded tissue were placed on glass slides, rehydrated, incubated with 3% hydrogen peroxide to quench endogenous peroxidase activity, and then blocked in 3% BSA/PBS. Sections were incubated with the primary mouse polyclonal anti-human WISP-1 and VEGF-A antibodies at a 1:50 dilution and incubated at 4°C overnight. After three PBS washes, sections were incubated with a 1:50 dilution of biotin-labeled goat anti-mouse IgG secondary antibody. Bound antibodies were detected using the ABC Kit (Vector Laboratories, Burlingame, CA, USA). Slides were stained with the chromogen diaminobenzidine, washed, counterstained with Delafield's hematoxylin, dehydrated, treated with xylene, and mounted.

For the WISP-1, VEGF-A, and CD31 IHC assays on the *in vivo* xenograft model tissues, and plug, tumor samples collected from euthanized mice were fixed in 4% paraformaldehyde in PBS for at least 72 h, dehydrated in increasing concentrations of ethanol, then embedded in paraffin. Serial sections of 5-μm thickness were cut longitudinally and incubated with anti-WISP-1 (1:50), anti-VEGF-A (1:50), or anti-CD31 antibody (1:50) at 4°C overnight. After three PBS washes, sections were incubated in a 1:50 dilution of biotin-labeled goat anti-mouse IgG secondary antibody, and bound antibodies were detected using the ABC Kit. The slides were stained with chromogen diaminobenzidine, washed, counterstained with Delafield's hematoxylin, dehydrated, treated with xylene, and mounted.

### Statistical analysis

Data are presented as the mean ± standard error of the mean (SEM). Statistical comparisons between 2 samples were performed using the Student's *t*-test. Statistical comparisons of more than 2 groups were performed using one-way analysis of variance with Bonferroni's post-hoc test. A *P*-value of less than 0.05 was considered statistically significant.

## SUPPLEMENTARY METHODS, FIGURES



## Abbreviations

OSCC: Oral squamous cell carcinomas
VEGF-A: vascular endothelial growth factor-A
EPCs: Endothelial progenitor cells
WISP-1/CCN4: WNT1-inducible signaling pathway protein 1
FAK: focal adhesion kinase
EGFR: epidermal growth factor receptor
ERK: extracellular signal-regulated kinase
HIF-1α: hypoxia inducible factor 1-α

## References

[1] Warnakulasuriya S (2009). Global epidemiology of oral and oropharyngeal cancer. Oral Oncol.

[2] Johnson NW, Jayasekara P, Amarasinghe AA (2011). Squamous cell carcinoma and precursor lesions of the oral cavity: epidemiology and aetiology. Periodontol 2000.

[3] Johnson NW, Warnakulasuriya S, Gupta PC, Dimba E, Chindia M, Otoh EC, Sankaranarayanan R, Califano J, Kowalski L (2011). Global oral health inequalities in incidence and outcomes for oral cancer: causes and solutions. Adv Dent Res.

[4] Kim SY, Nam SY, Choi SH, Cho KJ, Roh JL (2011). Prognostic value of lymph node density in node-positive patients with oral squamous cell carcinoma. Ann Surg Oncol.

[5] Bock JM, Sinclair LL, Bedford NS, Jackson RE, Lee JH, Trask DK (2008). Modulation of cellular invasion by VEGF-C expression in squamous cell carcinoma of the head and neck. Arch Otolaryngol Head Neck Surg.

[6] Eckert AW, Lautner MH, Schutze A, Bolte K, Bache M, Kappler M, Schubert J, Taubert H, Bilkenroth U (2010). Co-expression of Hif1alpha and CAIX is associated with poor prognosis in oral squamous cell carcinoma patients. J Oral Pathol Med.

[7] Lin PY, Yu CH, Wang JT, Chen HH, Cheng SJ, Kuo MY, Chiang CP (2008). Expression of hypoxia-inducible factor-1 alpha is significantly associated with the progression and prognosis of oral squamous cell carcinomas in Taiwan. J Oral Pathol Med.

[8] Brennan PA, Mackenzie N, Quintero M (2005). Hypoxia-inducible factor 1alpha in oral cancer. J Oral Pathol Med.

[9] Eckert AW, Kappler M, Schubert J, Taubert H (2012). Correlation of expression of hypoxia-related proteins with prognosis in oral squamous cell carcinoma patients. Oral Maxillofac Surg.

[10] Kang FW, Gao Y, Que L, Sun J, Wang ZL (2013). Hypoxia-inducible factor-1alpha overexpression indicates poor clinical outcomes in tongue squamous cell carcinoma. Exp Ther Med.

[11] Maeda T, Matsumura S, Hiranuma H, Jikko A, Furukawa S, Ishida T, Fuchihata H (1998). Expression of vascular endothelial growth factor in human oral squamous cell carcinoma: its association with tumour progression and p53 gene status. J Clin Pathol.

[12] Mineta H, Miura K, Ogino T, Takebayashi S, Misawa K, Ueda Y, Suzuki I, Dictor M, Borg A, Wennerberg J (2000). Prognostic value of vascular endothelial growth factor (VEGF) in head and neck squamous cell carcinomas. Br J Cancer.

[13] Holbourn KP, Acharya KR, Perbal B (2008). The CCN family of proteins: structure-function relationships. Trends Biochem Sci.

[14] Perbal B (2001). NOV (nephroblastoma overexpressed) and the CCN family of genes: structural and functional issues. Mol Pathol.

[15] Kleer CG, Zhang Y, Pan Q, Merajver SD (2004). WISP3 (CCN) is a secreted tumor-suppressor protein that modulates IGF signaling in inflammatory breast cancer. Neoplasia.

[16] Kleer CG, Zhang Y, Pan Q, van Golen KL, Wu ZF, Livant D, Merajver SD (2002). WISP3 is a novel tumor suppressor gene of inflammatory breast cancer. Oncogene.

[17] Xu L, Corcoran RB, Welsh JW, Pennica D, Levine AJ (2000). WISP-1 is a Wnt-1- and beta-catenin-responsive oncogene. Genes Dev.

[18] Pennica D, Swanson TA, Welsh JW, Roy MA, Lawrence DA, Lee J, Brush J, Taneyhill LA, Deuel B, Lew M, Watanabe C, Cohen RL, Melhem MF, Finley GG, Quirke P, Goddard AD (1998). WISP genes are members of the connective tissue growth factor family that are up-regulated in wnt-1-transformed cells and aberrantly expressed in human colon tumors. Proc Natl Acad Sci U S A.

[19] Chen PP, Li WJ, Wang Y, Zhao S, Li DY, Feng LY, Shi XL, Koeffler HP, Tong XJ, Xie D (2007). Expression of Cyr61, CTGF, and WISP-1 correlates with clinical features of lung cancer. PLoS One.

[20] Xie D, Yin D, Wang HJ, Liu GT, Elashoff R, Black K, Koeffler HP (2004). Levels of expression of CYR61 and CTGF are prognostic for tumor progression and survival of individuals with gliomas. Clin Cancer Res.

[21] Bergers G, Benjamin LE (2003). Tumorigenesis and the angiogenic switch. Nat Rev Cancer.

[22] Chuang JY, Chang AC, Chiang IP, Tsai MH, Tang CH (2013). Apoptosis signal-regulating kinase 1 is involved in WISP-1-promoted cell motility in human oral squamous cell carcinoma cells. PLoS One.

[23] Brigstock DR (2002). Regulation of angiogenesis and endothelial cell function by connective tissue growth factor (CTGF) and cysteine-rich 61 (CYR61). Angiogenesis.

[24] Lin CG, Leu SJ, Chen N, Tebeau CM, Lin SX, Yeung CY, Lau LF (2003). CCN3 (NOV) is a novel angiogenic regulator of the CCN protein family. J Biol Chem.

[25] Gao D, Nolan D, McDonnell K, Vahdat L, Benezra R, Altorki N, Mittal V (2009). Bone marrow-derived endothelial progenitor cells contribute to the angiogenic switch in tumor growth and metastatic progression. Biochim Biophys Acta.

[26] Wu CL, Tsai HC, Chen ZW, Wu CM, Li TM, Fong YC, Tang CH (2013). Ras activation mediates WISP-1-induced increases in cell motility and matrix metalloproteinase expression in human osteosarcoma. Cell Signal.

[27] Mitra SK, Schlaepfer DD (2006). Integrin-regulated FAK-Src signaling in normal and cancer cells. Curr Opin Cell Biol.

[28] Moro L, Venturino M, Bozzo C, Silengo L, Altruda F, Beguinot L, Tarone G, Defilippi P (1998). Integrins induce activation of EGF receptor: role in MAP kinase induction and adhesion-dependent cell survival. EMBO J.

[29] Taniguchi K, Xia L, Goldberg HJ, Lee KW, Shah A, Stavar L, Masson EA, Momen A, Shikatani EA, John R, Husain M, Fantus IG (2013). Inhibition of Src kinase blocks high glucose-induced EGFR transactivation and collagen synthesis in mesangial cells and prevents diabetic nephropathy in mice. Diabetes.

[30] Hsieh HL, Sun CC, Wang TS, Yang CM (2008). PKC-delta/c-Src-mediated EGF receptor transactivation regulates thrombin-induced COX-2 expression and PGE(2) production in rat vascular smooth muscle cells. Biochim Biophys Acta.

[31] Schachtrup C, Lu P, Jones LL, Lee JK, Lu J, Sachs BD, Zheng B, Akassoglou K (2007). Fibrinogen inhibits neurite outgrowth via beta 3 integrin-mediated phosphorylation of the EGF receptor. Proc Natl Acad Sci U S A.

[32] PO DEL, Jorge CC, Oliveira DT, Pereira MC (2014). Hypoxic condition and prognosis in oral squamous cell carcinoma. Anticancer Res.

[33] Ahluwalia A, Tarnawski AS (2012). Critical role of hypoxia sensor–HIF-1alpha in VEGF gene activation. Implications for angiogenesis and tissue injury healing. Curr Med Chem.

[34] Welti J, Loges S, Dimmeler S, Carmeliet P (2013). Recent molecular discoveries in angiogenesis and antiangiogenic therapies in cancer. J Clin Invest.

[35] Chen CC, Lau LF (2009). Functions and mechanisms of action of CCN matricellular proteins. Int J Biochem Cell Biol.

[36] Hodivala-Dilke K (2008). alphavbeta3 integrin and angiogenesis: a moody integrin in a changing environment. Curr Opin Cell Biol.

[37] Hodivala-Dilke KM, Reynolds AR, Reynolds LE (2003). Integrins in angiogenesis: multitalented molecules in a balancing act. Cell Tissue Res.

[38] Mitra SK, Lim ST, Chi A, Schlaepfer DD (2006). Intrinsic focal adhesion kinase activity controls orthotopic breast carcinoma metastasis via the regulation of urokinase plasminogen activator expression in a syngeneic tumor model. Oncogene.

[39] Mitra SK, Mikolon D, Molina JE, Hsia DA, Hanson DA, Chi A, Lim ST, Bernard-Trifilo JA, Ilic D, Stupack DG, Cheresh DA, Schlaepfer DD (2006). Intrinsic FAK activity and Y9 phosphorylation facilitate an angiogenic switch in tumors. Oncogene.

[40] Greenblatt M, Shubi P (1968). Tumor angiogenesis: transfilter diffusion studies in the hamster by the transparent chamber technique. J Natl Cancer Inst.

[41] Ehrmann RL, Knoth M (1968). Choriocarcinoma. Transfilter stimulation of vasoproliferation in the hamster cheek pouch. Studied by light and electron microscopy. J Natl Cancer Inst.

[42] Sharma M, Sah P, Sharma SS, Radhakrishnan R (2013). Molecular changes in invasive front of oral cancer. J Oral Maxillofac Pathol.

[43] Dornhofer N, Spong S, Bennewith K, Salim A, Klaus S, Kambham N, Wong C, Kaper F, Sutphin P, Nacamuli R, Hockel M, Le Q, Longaker M, Yang G, Koong A, Giaccia A (2006). Connective tissue growth factor-specific monoclonal antibody therapy inhibits pancreatic tumor growth and metastasis. Cancer Res.

[44] Aikawa T, Gunn J, Spong SM, Klaus SJ, Korc M (2006). Connective tissue growth factor-specific antibody attenuates tumor growth, metastasis, and angiogenesis in an orthotopic mouse model of pancreatic cancer. Mol Cancer Ther.

[45] Wang HH, Lin CA, Lee CH, Lin YC, Tseng YM, Hsieh CL, Chen CH, Tsai CH, Hsieh CT, Shen JL, Chan WH, Chang WH, Yeh HI (2011). Fluorescent gold nanoclusters as a biocompatible marker for *in vitro* and *in vivo* tracking of endothelial cells. ACS Nano.

[46] Xia X, Lemieux ME, Li W, Carroll JS, Brown M, Liu XS, Kung AL (2009). Integrative analysis of HIF binding and transactivation reveals its role in maintaining histone methylation homeostasis. Proc Natl Acad Sci U S A.

[47] Nickols NG, Jacobs CS, Farkas ME, Dervan PB (2007). Modulating hypoxia-inducible transcription by disrupting the HIF-1-DNA interface. ACS Chem Biol.

