# Supplementary material for: WISP-1, a novel angiogenic regulator of the CCN family, promotes oral squamous cell carcinoma angiogenesis through VEGF-A expression
Source: Oncotarget. 2015 Jan 30;6(6):4239–52. doi: 10.18632/oncotarget.2978 (PMC4414186; doi:10.18632/oncotarget.2978)
Supplement: Supplementary file 1 [file oncotarget-06-4239-s001.pdf]

SUPPLEMENTARY METHODS

Western blot

Cell lysates were prepared. Proteins were resolved on SDS-PAGE and transferred to an Immobilon polyvinyldifluoride (PVDF) membrane (EMD Millipore, MA, USA). Blots were blocked with 4% bovine serum albumin (BSA) for 1 h at room temperature and then probed with antibodies (1:1000) for 1 h at room temperature. After three washes, blots were incubated with a peroxidase-conjugated secondary antibody (1:1000) for 1 h at room temperature and visualized by enhanced chemiluminescence, using X-OMAT LS film (Eastman Kodak, Rochester, NY, USA). Data were quantified using a computing densitometer and ImageQuant software (Molecular Dynamics, Sunnyvale, CA, USA).

Quantitative real-time polymerase chain reaction (PCR)

qPCR analysis was performed using a Taqman® one-step PCR Master Mix (Applied Biosystems, Foster City, CA, USA). Total cDNA (100 ng) was added to sequence-specific primers and Taqman® probes. The final reaction volume was 25 µL. All target gene primers and probes were purchased, β-actin was used as an internal control (Applied Biosystems). The qPCR assay was run in triplicate using the StepOnePlus sequence detection system (Life Technologies, CA, USA). Cycling conditions were as follows: polymerase activation at 95°C for 10 min followed by 40 cycles at 95°C for 15 s and 60°C for 60 s. The threshold was set above the non-template control background and within the linear phase of the target gene amplification to calculate the cycle number at which the transcript was detected (denoted as CT).

Transwell migration assay

This process used transwell inserts (8-µm pore size; Costar, NY, USA) in 24-well plates. OSCC cells were pretreated for 30 min with designated inhibitors or the vehicle (0.1% dimethyl sulfoxide (DMSO)). Alternatively, OSCC cells were transfected with the indicated siRNAs for 24 h, and the conditioned medium (CM) was collected after 24 h. EPCs were seeded in the upper transwell chamber and 300 µL of CM were placed in the lower chamber. After 20 h, the migration cells were stained with crystal violet and counted with microscope.

Enzyme-linked immunosorbent assay (ELISA)

OSCC cells were pretreated for 30 min with the designated inhibitors or the vehicle concentration (0.1% DMSO). Alternatively, OSCC cells were transfected with the indicated siRNAs for 24 h. CM was collected 24 h after transfection and stored at –80°C. VEGF-A secretion was measured using an ELISA kit (Biocompare, San Jose, CA, USA), as per the manufacturer's instructions.

Tube formation

Matrigel (BD Biosciences, Bedford, MA, USA) was dissolved at 4°C, and 150 µL aliquots were added to each well of 48-well plates, which were incubated at 37°C for 30 min. EPCs were resuspended at a density of 5×10^4/100 µL in culture medium (50% EGM-MV2 medium and 50% OSCC cell CM) and added to the wells. VEGF-A (20 ng/mL) and culture medium were used as positive and negative controls, respectively. After 6 h of incubation at 37°C, EPC tube formation was assessed by microscopy, and each well was photographed. The number of tube branches and total tube length were calculated using the MacBiophotonics Image J software.

Chick chorioallantoic membrane (CAM) assay

The angiogenic activity was evaluated using a CAM assay. Briefly, fertilized chicken eggs (5 eggs/group) were incubated at 37°C in an 80% humidified atmosphere. On developmental day 8, Matrigel mixed with CM from control-shRNA SCC4 cells or WISP-1 shRNA SCC4 cells were resuspended in Matrigel and placed onto CAMs for 4 days. The CAMs were then examined by microscopy and photographed. Angiogenesis was quantified by counting the number of blood vessel branches. All animal work was performed in accordance with a protocol approved by the China Medical University (Taichung, Taiwan) Institutional Animal Care and Use Committee.

Establishment of the WISP-1 knockdown SCC4 cell line

To establish the WISP-1 knockdown SCC4 cell line, lentiviral vectors for expression of the CCN3-specific shRNAs or a negative control (Neg) shRNA were purchased from the National RNAi Core Facility Platform (Taipei, Taiwan). Lentiviruses were prepared according to standard protocols. For infection, SCC4 cells were seeded in a 6-well dish and the lentivirus was added (multiplicity of infection = 10) in medium containing polybrene (8 µg/ml). After 24 h, the culture medium was changed, and 48 h later, 1 mg/ml puromycin was added to select for Neg shRNA- and WISP-1 shRNA-expressing cells. The surviving cells were selected and clonal cell populations were expanded. For monolayer growth curves, 10^3 cells were plated in 6 well plates and grown for 1 to 3 days. Cells were trypsinized, and cell numbers was counted.

SUPPLEMENTARY FIGURES

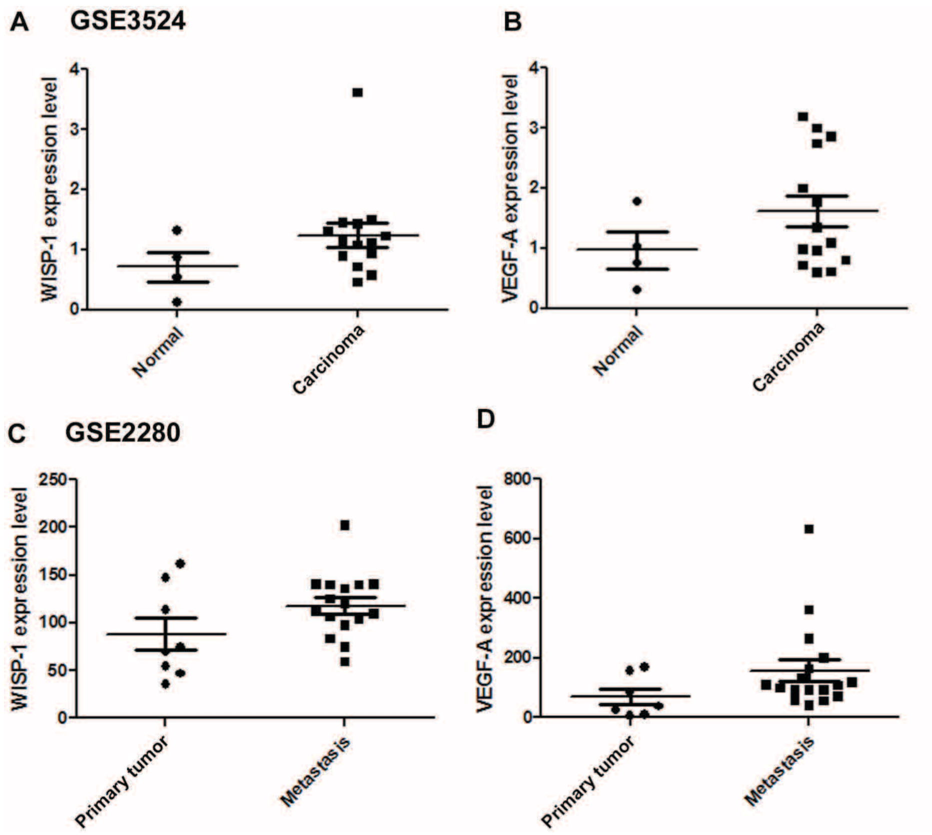

Supplementary Figure S1: Higher expression levels of WISP-1 and VEGF-A in OSCC tumor specimens. mRNA expression levels of WISP-1 and VEGF-A in specimens from patients with OSCC microarray datasets GSE3524 **(A–B)** and GSE2280 **(C–D)** Data are expressed as the mean ± SEM **P* < 0.05 compared with the normal tissues group (A–B) and primary tumor specimens group (C–D).

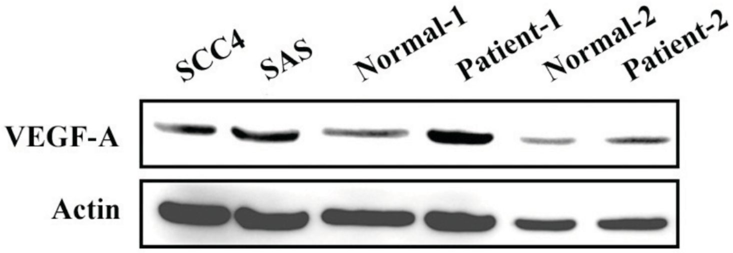

Supplementary Figure S2: Protein expression is higher in OSCC cell lines (SCC4, SAS) and tumor specimens compared with normal specimens. Total cell lysate were prepared from OSCC cell lines (SCC4, SAS), tumor specimens and paired adjacent normal specimens. Western blot were performed to detect VEGF-A expression. Actin was used as control.

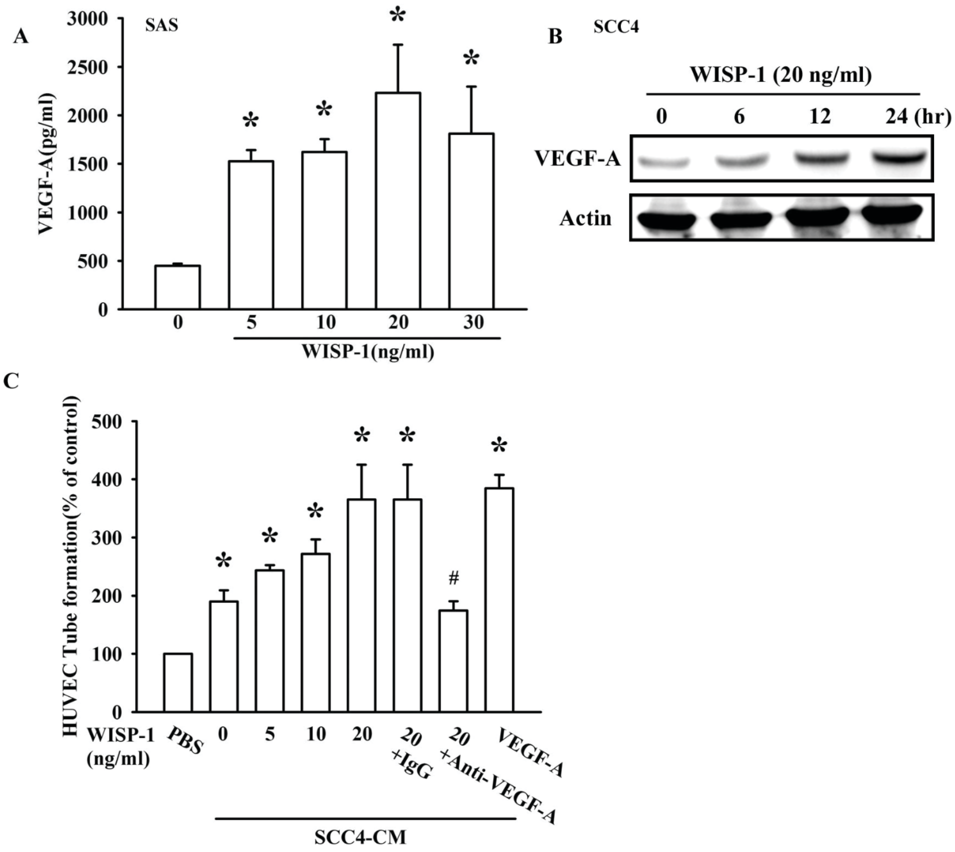

Supplementary Figure S3: WISP-1 regulates the angiogenesis by raising VEGF-A expression in OSCC cells. **(A)** SCC4 cells were incubated with WISP-1 (20 ng/mL) for indicated times (0–24 h), VEGF-A expression was measured by ELISA. **(B)** SAS cells were incubated with WISP-1 (0–30 ng/mL) for 24 h, VEGF-A expression was measured by Western blot. **(C)** SCC4 cells were incubated with WISP-1 (0–20 ng/mL) for 24 h, and the CM was collected. HUVEC cells were pre-treated for 30 min with IgG control antibody or VEGF-A antibody (1 μg/mL) and incubated with CM for 6 h and cell capillary-like structure formation in HUVEC cells was examined by tube formation assay. Data are expressed as mean ± SEM $*P < 0.05$ compared to control. $\#P < 0.05$ compared to WISP-1 20 ng/ml treatment group.

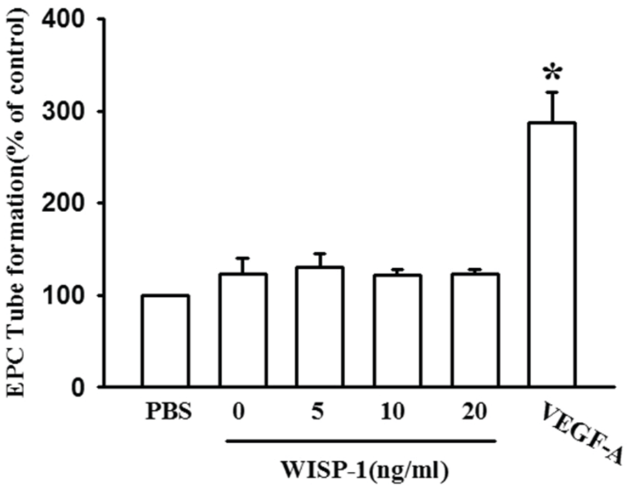

Supplementary Figure S4: WISP-1 alone has no angiogenic effect in EPCs. EPCs were incubated with indicated dose of WISP-1 (0–20 ng/ml) for 6 h and cell capillary-like structure formation in EPCs was examined by tube formation assay. VEGF was used as positive control. Data are expressed as mean ± SEM $*P < 0.05$ compared to PBS contorl.

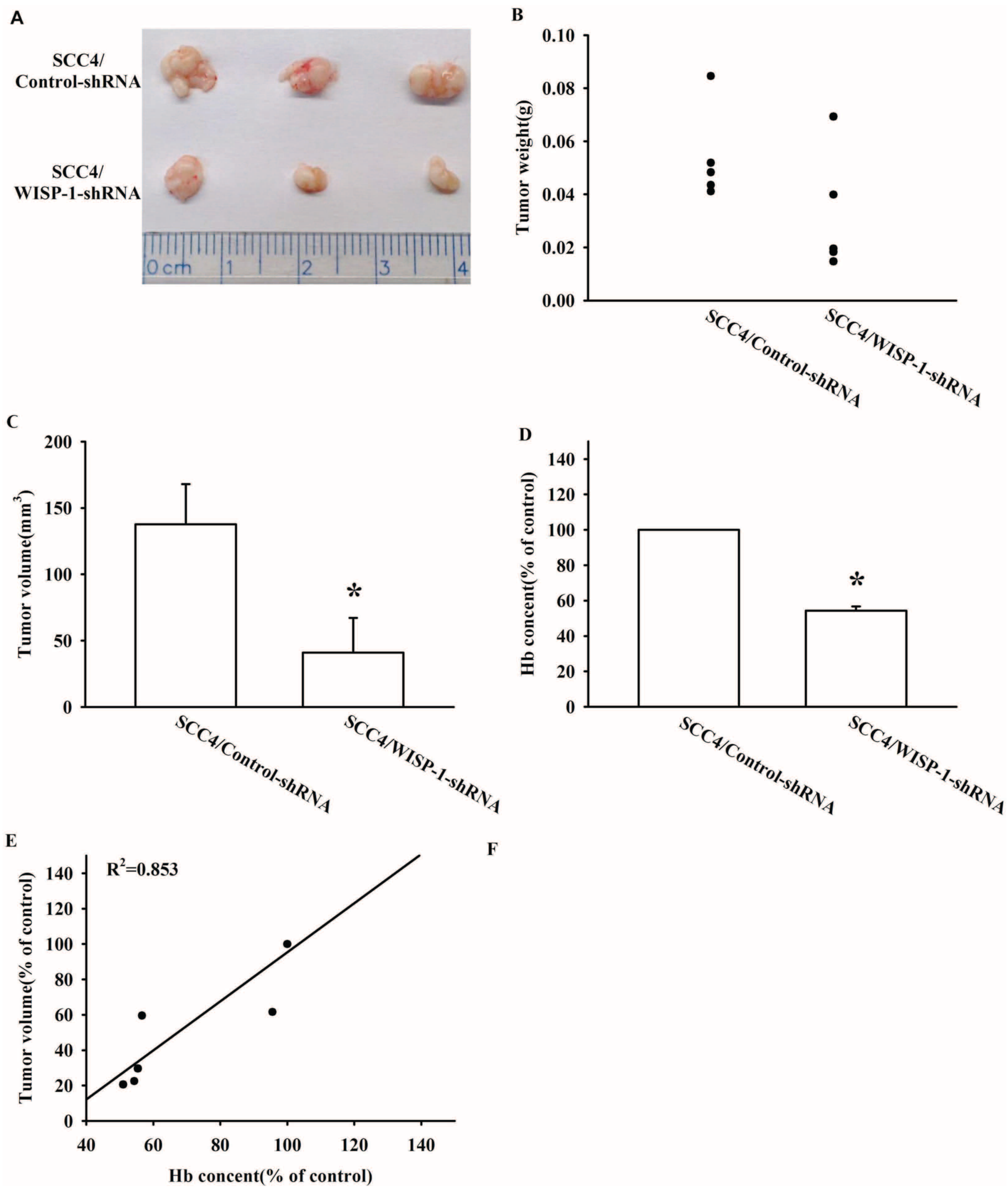

Supplementary Figure S5: WISP-1 knockdown decreases angiogenesis-related tumor growth *in vivo*. (A–D) Control shRNA and WISP-1 shRNA SCC4 cells were mixed with Matrigel and injected into the flank of the mice for 28 days, after which the mice were euthanized. Tumors were resected, photographed, weight and volume were measured, and hemoglobin was quantified. The correlation between tumor volume and hemoglobin level is presented in **(E)** Data are expressed as the mean ± SEM *$P < 0.05$ compared with the control shRNA/SCC4 group.

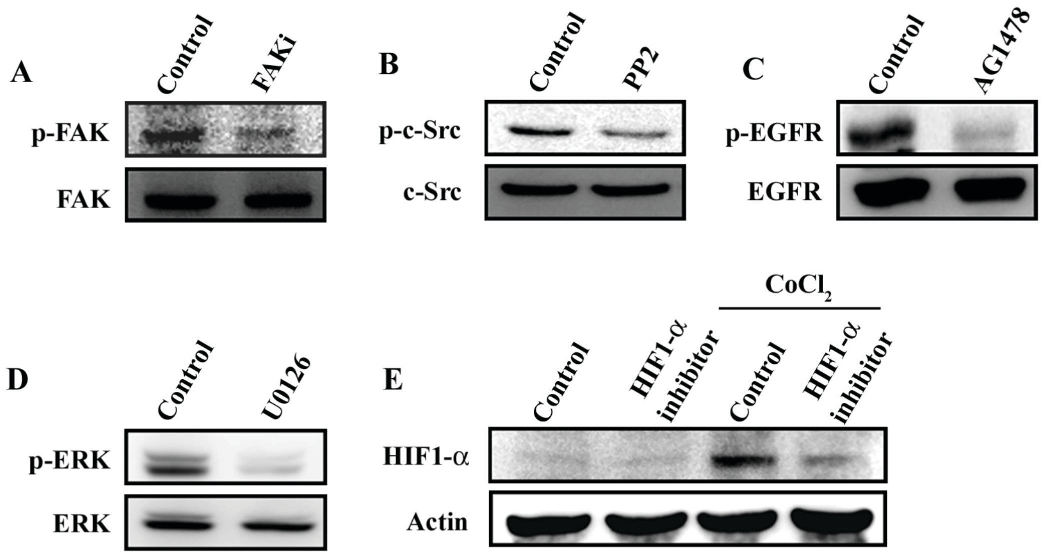

Supplementary Figure S6: The efficacy of all inhibitors which are used in this study. **(A–D)** SCC4 cells were incubated with the indicated inhibitors (FAKi, pp2, AG1478 and U0126) for 60 min. Cell lysates were collected and Western blot was performed to detect p-FAK, p-c-Src, p-EGFR and p-ERK. **(E)** SCC4 cells were pretreated with HIF1-α inhibitor for 30 min then incubated with $CoCl_2$ (a chemical inducer of hypoxia) for 4 hr. Cell lysates were collected and Western blot was performed to detect HIF1-α accumulation.
